# Saliva tau and phospho-tau-181 measured by Lumipulse in patients with Alzheimer’s disease

**DOI:** 10.3389/fnagi.2022.1014305

**Published:** 2022-09-29

**Authors:** Josef Marksteiner, Michaela Defrancesco, Christian Humpel

**Affiliations:** ^1^Department of Psychiatry and Psychotherapy A, State Hospital Hall in Tirol, Hall in Tirol, Austria; ^2^Division of Psychiatry I, Department of Psychiatry, Psychotherapy, Psychosomatics and Medical Psychology, Medical University Innsbruck, Innsbruck, Austria; ^3^Laboratory of Psychiatry and Experimental Alzheimer’s Research, Medical University of Innsbruck, Innsbruck, Austria

**Keywords:** Alzheimer’s disease, saliva, biomarker, tau, beta-amyloid

## Abstract

Alzheimer’s disease (AD) is a severe neurodegenerative brain disorder. The determination of beta-amyloid (Aβ)-40, –42, total tau, and phospho-tau-181 (pTau181) in cerebrospinal fluid (CSF) using Lumipulse technology has been established as biomarkers for AD in recent years. As CSF collection is an invasive procedure, one aims to find biomarkers in blood or other human fluids, such as saliva. In the present study, we aim to measure these markers in human saliva. Using Salivettes, we collected saliva samples from healthy controls (*n* = 27), patients with AD dementia (*n* = 44), mild cognitive impairment (MCI) (*n* = 45), depression (*n* = 31), and 21 blinded samples, all older than 60 years. Lumipulse technology with a G600II was used to detect all four biomarkers. Our data show that the levels of total protein were highly variable and thus biomarker levels were corrected to 1 mg/ml of total protein. Saliva Aβ−40 and –42 were not detectable, because it was not recovered from the Salivettes. However, saliva total tau (577 ± 134 pg/mg, *n* = 22) and phospho-tau-181 (9.7 ± 1.3 pg/mg, *n* = 21) could be analyzed by Lumipulse technology. Saliva total tau levels were significantly decreased in patients with AD (≤ 300 pg/mg protein), while pTau181 levels (≥ 18 pg/mg protein) were significantly enhanced in patients with MCI compared to controls. Laboratory diagnosis with a cut-off of ≥ 18 pg/mg protein pTau181 (for MCI) and ≤ 300 pg/mg protein tau (for AD) for blinded samples could diagnose MCI and AD with an accuracy of 71.4%. Despite these initial promising results, the findings must be replicated in larger cohorts, and several technical problems due to saliva processing must be solved and Salivettes should not be used.

## Introduction

The concept of Alzheimer’s disease (AD) has extensively changed over the past years and there is increasing evidence for a long preclinical disease stage with hardly any clinical and cognitive impairment but detectable microstructural changes in the brain and metabolic changes in body fluids. Following a preclinical phase, AD patients merge to mild cognitive impairment stage (MCI) and finally convert to clinically manifest AD dementia (Sperling et al., 2011). Within the last century, the mean life expectancy of humans has increased from around 40 to approximately 80 years. As age is the main risk factor for AD, the number of patients suffering from AD will dramatically increase within the next 50 years so about 80 million patients with AD in the dementia stage can be expected worldwide by 2050. These enormously high numbers of presumed patients with AD dementia call for further establishment of reliable diagnostic surrogate markers for diagnosing and monitoring disease progression and therapy. A valid and easily accessible diagnostic procedure should be the basis for treatment. Definitive diagnosis of AD requires both a clinical diagnosis and post-mortem detection of AD pathologies. A probable diagnosis of AD or prediction of imminent conversion from MCI to AD dementia can be made based on clinical criteria, laboratory tests, neuroimaging, and neuropsychological evaluation with moderate certainty depending on available diagnostic possibilities.

A promising area of research for laboratory diagnosis of AD is the analysis of cerebrospinal fluid (CSF), where the measurement of beta-amyloid (Aβ) with 40 or 42 amino acids, total tau, and phospho-tau-181 can distinguish patients with AD from healthy subjects with high specificity and sensitivity (Blennow, 2005; Blennow et al., 2010; Humpel, 2011). Further on, numerous studies have found abnormalities of the noradrenergic system with higher levels of CSF norepinephrine (NE) in patients with AD (Sheline et al., 1998), and lower levels of plasma NE in patients with depression (Anand and Charney, 2000). The latter is a well-known risk factor for AD (Defrancesco et al., 2017). Unfortunately, the use of CSF biomarkers is limited by invasive collection. Non-invasive methods for the detection of cerebral Aβ and tau, e.g., by positron emission tomography have the disadvantage of very high costs and lack of availability.

Thus, there is a need to discover biomarkers in other human biological fluids, such as blood, urine, or saliva, which are easily obtainable and allow collecting a high number of samples. In 2008, Li et al. (2008) showed a relationship between saliva levels of 3-methoxy-4-hydroxyphenylglycol (sMHPG) - a NE metabolite, and mental health in the elderly general population. In the same year, Boston et al. (2008) developed a simple laboratory test to measure acetylcholinesterase as a possible biomarker for AD in saliva. Another pilot study reports on the discovery of diagnostic biomarkers in saliva of patients with AD using 1H-NMR-based metabolomics (Yilmaz et al., 2017). Recently we (Marksteiner et al., 2019) have shown for the first time using targeted metabolomics that acyl-alkyl phosphatidylcholines (sum of PCae C34:1-2; PCae C36:1-2-3; PCaeC38:1-3; PCae C40:2-3) are significantly reduced in saliva of patients with AD dementia compared to healthy controls. Thus, saliva could serve as a very powerful and easily accessible human fluid to detect biomarkers of AD (Huan et al., 2018; Ashton et al., 2019; François et al., 2019; Gleerup et al., 2019; Liang and Lu, 2019).

Regarding the expression of the Aβ and tau AD biomarkers in saliva, only very few data have been published and some are not clear and controversial. In 2010, Bermejo-Pareja et al. (2010) reported for the first time that saliva Aβ−42 could become a potential biomarker, which was recently reproduced by others (Lee et al., 2017; Sabbagh et al., 2018; Gleerup et al., 2019). There is preliminary evidence that also tau is found in saliva. In 2011, Shi et al. (2011) reported the presence of tau and phospho-tau in saliva confirmed by mass-spectrometry. However, there are many discrepancies and so far the role of tau in saliva is not clear. Some authors found that Aβ−42 is enhanced in saliva (Bermejo-Pareja et al., 2010), while others could not detect it (Shi et al., 2011). Some authors show that saliva tau is not associated with AD (Ashton et al., 2018), while others see an increase in early AD (Bermejo-Pareja et al., 2010). In CSF, normal levels of phospho-tau-181 (< 60 pg/ml) are markedly lower than those of total tau (< 500 pg/ml). In saliva, tau levels are low (20 pg/ml) but others found 3–5x higher phospho-tau levels (Lau et al., 2015; Tvarijonaviciute et al., 2020). Meanwhile, tau saliva has also been detected in relapsing-remitting multiple sclerosis (Mirzaii-Dizgah et al., 2020) and traumatic brain injury (Olczak et al., 2019).

Tau is a microtubule-associated protein and physiologically stabilizes and regulates axonal transport (Liu and Götz, 2013; Spillantini and Goedert, 2013; Nisbet et al., 2015; Wang and Mandelkow, 2016). However, the physiological roles of tau seem to be more complex and by far not fully explored. Full-length tau (2N4R) exists also in truncated forms (1N4R, 0N4R, 2N3R, 1N3R, and 0N3R) and tau has more than 40 possible phosphorylation sites (Grundke-Iqbal et al., 1986; Hanger et al., 2009). The phosphorylation is regulated by several kinases and phosphatases and it is extremely important to identify these critical phosphorylation sites in the tau protein, as they are either therapeutic or diagnostic targets (Liu and Götz, 2013; Spillantini and Goedert, 2013; Wang and Mandelkow, 2016). In our laboratory, we have extensive routine experience in the analysis of tau and pTau181 in CSF using Lumipulse (Blasko et al., 2006; Lederer et al., 2016), but the functional role of tau in saliva is completely unknown.

In this study, we aim to analyze Aβ–42 and –40, total tau, and pTau181 in saliva collected with Salivettes. We investigate whether there are disease-specific changes in cognitively healthy subjects, patients with MCI, AD dementia, or depression using the well-established Lumipulse technology. The purpose of the study was to determine possible biomarkers for diagnosing clinical and preclinical stages of AD in saliva.

## Materials and methods

### Patients

Cognitively healthy subjects and patients suffering from AD, MCI, and depression were recruited at the state hospital Hall/Tirol, as reported in detail in several previous studies (Marksteiner et al., 2019). The following groups were included in this study: healthy controls (group 1, *n* = 27), patients with MCI (group 2, *n* = 45), patients with AD dementia (group 3, *n* = 44), and depressive patients without marked cognitive impairment (group 4, *n* = 31). For the blinded study another 21 saliva samples from healthy controls and patients with AD dementia or MCI aged older than 60 years were collected at the memory clinic of the University Clinic of Psychiatry Innsbruck in July 2022. All patients completed a clinical and neuropsychological assessment including subtests of the “Consortium to Establish a Registry for Alzheimer’s Disease” (CERAD) battery (Rosen et al., 1984) as well as the MMSE (Folstein et al., 1975). The MMSE served as a measure for the severity of cognitive impairment. The 15 items version geriatric depression scale (GDS-15) was used to assess depressive symptoms. Cerebral neuroimaging (magnetic resonance imaging, MRI) was performed to evaluate cortical atrophy, cerebrovascular pathology, and to exclude other brain pathologies. Probable AD dementia was diagnosed according to the current NINCDS-ARDRA (National Institute of Neurological and Communicative Disorders and Stroke and the AD and Related Disorders Association) criteria. MCI was diagnosed according to the criteria of Petersen et al. (2001). A general blood examination was part of the routine diagnostic procedure. Exclusion criteria for healthy subjects, patients with MCI, and AD dementia included (1) another primary neurological or mental disorder, (2) any kind of metabolic decompensation or any sign of peripheral inflammation (e.g., rheumatic disease), (3) long-term alcohol or drug abuse, (4) or any current, clinically significant cardiovascular disease, (5) or current intake of medication that could influence saliva production or saliva composition (e.g., anticholinergic medication, cyclosporine, immunosuppressives, bronchodilators, anti-inflammatory drugs). The study was approved by the ethics committee of the Medical University of Innsbruck. All subjects and/or their caregivers enrolled in the study gave their informed consent. In preliminary experiments, we analyzed tau and Aβ saliva levels in healthy volunteers aged from 5 to 90 years.

### Collection of saliva

While the total volume of saliva varies per individual, the average daily production ranges from 1 to 2 liters per day. Normal stimulated saliva flow is 1–2 ml per minute and rates below 0.6 ml/min are considered low. Collection of saliva is done in the early morning and all subjects were asked to refrain from eating, drinking, smoking, or using oral hygiene prior to saliva collection (at least for 8 h). We routinely used a Salivette (Sarstedt; Nr. 51.1534, Cotton). Patients were asked to have the cotton in the mouth for exactly 2 min in order to measure salivary flow. Our data showed that we collected 1.52 ± 0.04 ml/2 min (*n* = 5) using our Salivette. The samples were sent to the laboratory at room temperature within 4 h, centrifuged (3,000 × g 5 min), the volume was measured and recorded and saliva was aliquoted (500 μl) and frozen at –80^°^C until analysis (for all samples from Table 1; stored from 2016 to 2022). For the blinded study, saliva of 21 patients was collected in July 2022 and analyzed within 4 h after collection (without freezing).

**TABLE 1:** Demographic data and biomarkers in saliva.

	Control	MCI	AD dementia	Depression
*n*	27	45	44	31
M/F	13/14	20/25	19/25	10/21
Age [years]	71 ± 1	74 ± 1	79 ± 1*	70 ± 2
MMSE	29.6 ± 0.09	27.5 ± 0.2*	25.6 ± 5***	28.6 ± 0.2
GDS	1.9 ± 0.3	3.2 ± 0.4*	2.8 ± 0.4	7.8 ± 0.8***
Protein [μg/ml]	1,558 ± 341 (361–8,269)	1,388 ± 131 (245–3,606)	1,817 ± 178, (157–5,407)	1,597 ± 217 (236–4,162)
Norepinephrine[ng/ml] *n* = 12	7.5 ± 4.2	6.6 ± 3.3	0.45 ± 0.31*	17.6 ± 8.1, *p* = 0.1
Total tau	577 ± 134 (22)	485 ± 88 (14)	260 ± 53 (24)*	583 ± 201 (14)
Phospho-tau-181	9.7 ± 1.3 (21)	31.0 ± 12 (25)*	22.5 ± 3.6 (25)	11.7 ± 2.0 (11)
Ratio (tau/pTau181)	78 ± 17 (21)	38 ± 11 (13)	41 ± 17 (24)	68 ± 21 (11)

Saliva was collected from healthy controls, patients with Alzheimer’s disease (AD) dementia, mild cognitive impairment (MCI), or depression (n gives the number of analyzed patients). The Mini-Mental State Examination (MMSE) and the geriatric depression scale (GDS) were analyzed. Total protein was measured by Bradford assay and is given in μg/ml; the range in parenthesis shows the high grade of diversity. Norepinephrine was measured by HPLC-EC and is given as ng/ml. The four AD biomarkers beta-amyloid (Aβ), total tau, and phospho-tau-181 were analyzed by Lumipulse technology and are given as pg/mg total protein (corrected for 1 mg/ml total protein). All values are mean ± SEM. Statistical analysis was performed by one-way ANOVA with a subsequent Fisher LSD post-hoc test where p ≤ 0.05 was significant (*p ≤ 0.05; ***p ≤ 0.001). Please note that beta-amyloid-40 and –42 were not detectable when using Salivettes, as it is suggested that the small peptides are not recovered from the cotton.

### Analysis of saliva

Total protein was determined in undiluted saliva using the Bradford assay. NE was measured using HPLC-EC as described by us (Hutter et al., 1996).

**FIGURE 1:**
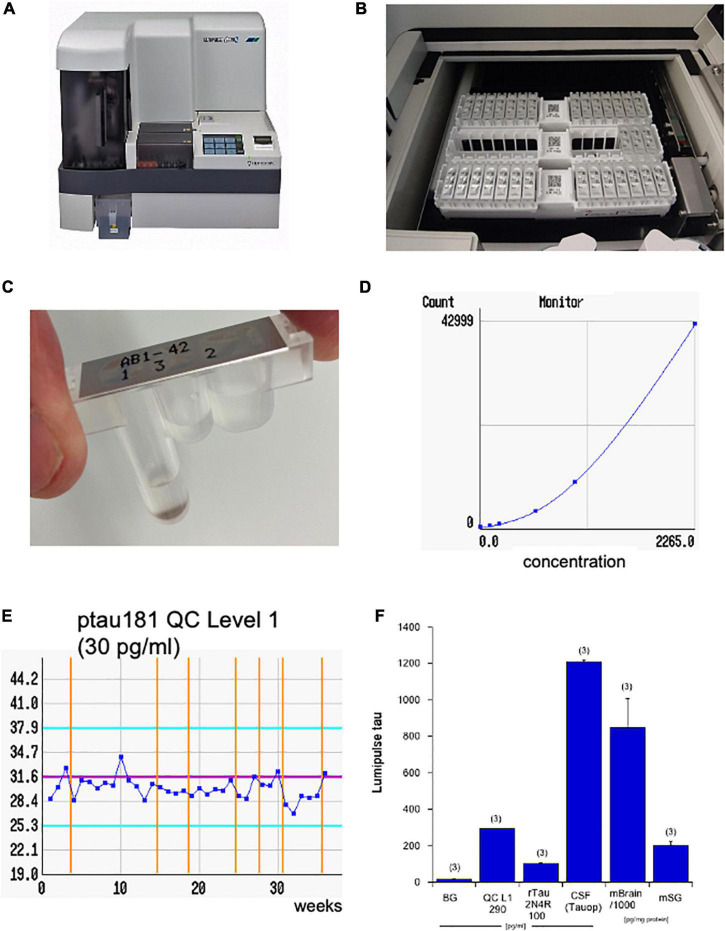
The four AD biomarkers were analyzed using the Lumipulse G600II **(A)**. The principle is an enzymatic reaction, where the antibodies and magnetic beads are stored in three different tubes within a single unit located on a rack with 14 units **(B,C)**. The computer generates a standard curve (**D**; e.g., for tau) and quality controls (QCs; e.g., for phospho-tau-181, 30 pg/ml) to guarantee an accurate analysis over weeks **(E)**. Values are generated from standards and display a very low background (BG) versus the tau quality level 1 (290 pg/ml), a recombinant 2N4R tau standard (100 pg/ml), as well as a cerebrospinal fluid (CSF) sample of a patient with tauopathy (Tauop) **(F)**. As a control tau levels are also shown in a mouse brain (mBrain) or in a mouse male salivary gland (mSG). The later ones are corrected for total protein and are given as pg/mg. Note that the brain contains extremely high tau values and are presented as 1,000× less in the diagram. Values are given as mean ± SEM, with *n* = 3 different samples.

Levels of Aβ–40, Aβ–42, total tau, and pTau181 were measured using automated Lumipulse enzymatic light emitting technology (Fujirebio G600II).^1^ The Lumipulse assay is an automated robotic platform (Figure 1A), using an enzymatic light emitting system. This system gives very fast and accurate values within 35 min. The single racks are placed in the system (Figure 1B) and each unit contains a triple-tube with the antibodies and magnetic beads (Figure 1C). For every analyte, a standard curve is generated (Figure 1D) and for every analyte also quality controls are run in parallel (Figure 1E). The background levels are very low (16 ± 3 pg/ml, *n* = 3), and every quality control (e.g., tau Level 1 290 = 292 ± 0.6 pg/ml, *n* = 3) or standard (e.g., full-length 2N4R tau 100 = 103 ± 4 pg/ml, *n* = 3) gives a very accurate reproducible value with very low variance (Figure 1F). As a control, a CSF sample (1,209 ± 8 pg/ml, analyzed as triplicate) is shown from a patient with tauopathy (Figure 1F). As an additional control, a wild-type mouse brain was analyzed giving very high tau levels of 847,000 ± 160,000 pg/mg total protein (*n* = 3) or a wild-type mouse male salivary gland gives very low tau levels of 201 ± 21 tau pg/mg protein (*n* = 3) (Figure 1F).

### Statistical analysis

Statistical analysis was performed by one-way ANOVA with a subsequent Fisher LSD *post-hoc* test where *p* ≤ 0.05 was significant. To determine differences between male and female participants (age-study), a student’s *t*-test was performed.

## Results

### Characteristics of the participants

In the present study, 147 subjects were included, which were grouped in healthy controls (*n* = 27, group 1), patients with MCI (*n* = 45, group 2), patients with AD dementia (*n* = 44, group 3), and patients with depression (*n* = 31, group 4) (Table 1). In all groups nearly 40% were male participant, only in the depressive group there were less male participant (Table 1). The mean age of the patients was 71 years, and only patients with AD dementia were slightly older (Table 1). For healthy subjects, the MMSE score was 29.6 ± 0.1 and was significantly lower in the MCI group and even lower in the AD dementia group (Table 1). Except in the depression group, the GDS score was lower than 5 (Table 1).

### Total protein and norepinephrine in saliva

**FIGURE 2:**
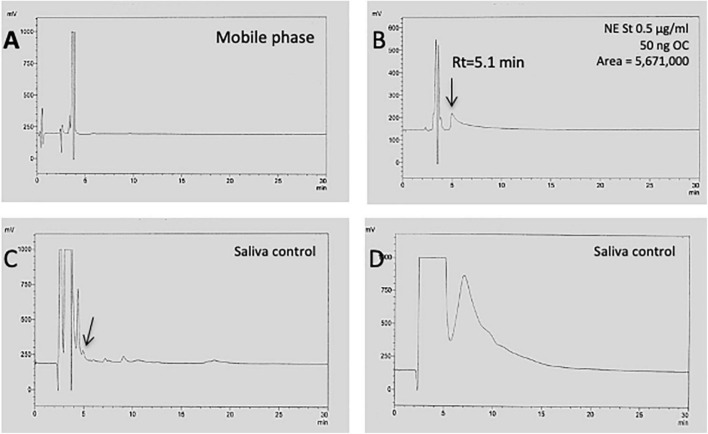
HPLC chromatogram of saliva to detect norepinephrine. When only the mobile phase was injected onto the HPLC, an injection peak was seen but only a very low background **(A)**. Injection of a norepinephrine (NE) standard (0.5 μg/ml) shows that NE elutes at approx. 5 min, giving a single peak **(B)**. Injection of pure saliva (1 mg/ml) shows a chromatogram with only a few peaks **(C)**, but also a very heterogeneous chromatogram, with a high peak load during the first 5 min, where NE could not be detected **(D)**.

In fresh saliva, the protein concentration was 1,558 ± 341 μg/ml in controls and was not significantly different from the other groups (Table 1). However, the variability of saliva total protein levels was high ranging from 0.16 mg/ml to 8.3 mg/ml (Table 1). This variability was also seen when saliva was analyzed by HPLC-EC detection. Norepinephrine (NE) concentration was determined as a measure of sympathetic neuronal activity indicating stress. Figure 2A shows a blank chromatogram and Figure 2B shows that a NE standard elutes at approx. 5 min. The detection limit was 100 pg on the column. The chromatogram for fresh saliva (diluted to 1 mg/ml) was very heterogeneous ranging from a few peaks (Figure 2C) to a very high protein load, where NE could not be detected (Figure 2D). The NE levels in control saliva were 7.5 ± 4.2 ng/ml and were not different in MCI, but significantly reduced in AD saliva and there was a tendency to be increased in depressive patients (Table 1).

### The four Alzheimer’s disease biomarkers in saliva

All four AD biomarkers were analyzed using the robotic-automated enzymatic Lumipulse assay (Figure 1). In order to exclude the heterogeneity of saliva samples, all values were corrected to 1 mg/ml total protein and are given as pg/mg total protein.

Beta-amyloid-40 was below the detection limit (5 pg/ml) in all cases but only two values were detectable in controls (5 and 20 pg/ml) and one value in depression (13 pg/ml). Beta-amyloid-42 was below the detection limit (9 pg/ml) in all cases, and only two values were detectable in controls (19 and 21 pg/ml). In order to exclude a stability problem over freezing at –80^°^C, saliva of seven healthy control individuals was tested fresh within 3 h and all seven samples of beta-amyloid-42 and –40 were below the detection level. In order to rule out that beta-amyloid is captured by the cotton in the Salivettes, we performed a recovery experiment. Our data show, indeed, that the recovery of beta-amyloid-40 from the Salivettes was very low (6.5 ± 3% recovery, *n* = 6). However, when native saliva was collected (only spitting in tubes, without Salivettes), the levels of beta-amyloid were clearly above the detection level: 89.5 ± 12.1 pg/ml (*n* = 4) Aβ(40) and 26.3 ± 5.1 pg/ml (*n* = 4) Aβ(42).

**FIGURE 3:**
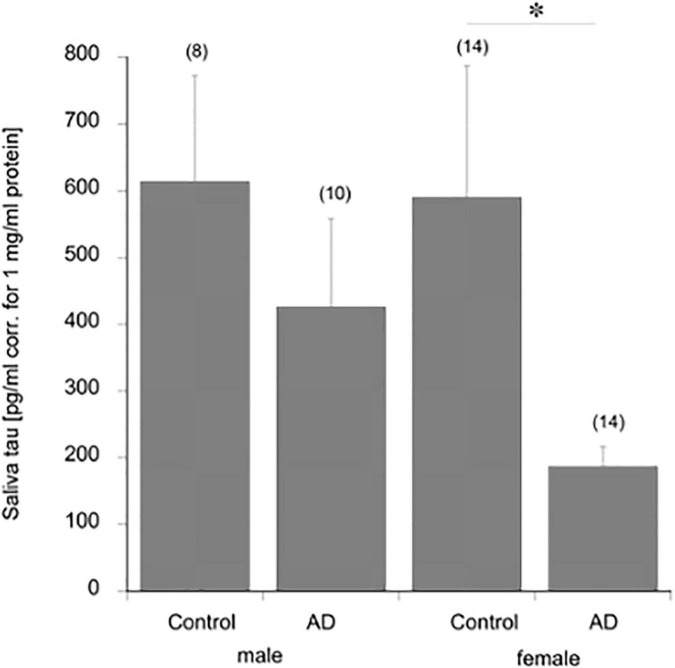
Total tau saliva levels in male and female controls and patients with Alzheimer’s disease dementia (AD). Saliva was collected, analyzed for total tau by Lumipulse, and corrected for the protein. Values are given as mean ± SEM and the values in parenthesis give the number of analyzed patients. Statistical analysis was performed by one-way ANOVA with a subsequent Fisher LSD *post-hoc* test (*p ≤ 0.05).

Total tau in saliva was 577 ± 134 pg/mg in healthy controls (Table 1). Tau levels did not differ significantly between male and female participants in controls (Figure 3). Total tau saliva levels were significantly reduced in patients with AD dementia (Table 1), but not in patients with MCI or depression (Table 1). This effect was markedly pronounced in female patients with AD dementia but not in male patients with AD dementia (Figure 3). The saliva levels of phospho-tau-181 were 9.7 ± 1.3 (*n* = 21) pg/mg in healthy controls (Table 1). The phospho-tau-181 saliva levels were significantly enhanced in patients with MCI (Table 1), but not in patients with depression (Table 1). There was a tendency for an increase in patients with AD dementia saliva (Table 1). The ratio tau/pTau181 did not provide a statistical significance. In a preliminary experiment, we analyzed saliva from healthy volunteer controls at an age between 5 and 90 years and found that saliva tau levels did not show an age-dependent effect (data not shown).

### Blinded study

In order to verify our values, we performed a blinded study (see Table 2). In this study, we collected 21 samples and determined a laboratory diagnosis with a cut-off of ≥ 18 pg/mg pTau181 (for MCI) and ≤ 300 pg/mg tau (for AD dementia). Out of these 21 samples, we excluded three with very high tau levels, one with very low protein, two with very high protein, and one after breakfast (Table 2). Out of the remaining 14 samples, we could diagnose 10 samples correctly (= 71.4%).

**TABLE 2:** Blinded verification of the diagnosis.

No	Gender	Age	pTau 181 [pg/mg protein] Cut-off for MCI ≥ 18	Tau [pg/mg protein] Cut-off for AD ≤ 300	Comment to exclude the sample	Laboratory diagnosis	Clinical diagnosis	Correct YES/NO
1	M	78	10	75,221	Extreme high tau	Unclear, CO	CO, parotitis	**X**
2	M	68	71	493		MCI	MCI/AD, MMSE 26	**Y**
3	M	68	4	3,106		CO	CO	**Y**
4	F	61	3	29	Very high protein (13 mg/ml)	Unclear, CO	CO	**X**
5	F	78	319	11,153	Extreme low protein (26 μg/ml)	Unclear, CO	MCI	**X**
6	M	85	30	223		MCI/ADD	MCI	**Y**
7	M	89	36	233		MCI/ADD	AD, MMSE 20	**Y**
8	M	77	15	338		CO	MCI	**N**
9	F	64	12	468		CO	CO	**Y**
10	F	68	79	949		MCI	CO	**N**
11	F	79	23	69		MCI/ADD	MCI	**Y**
12	M	74	63	122		MCI/ADD	MCI	**Y**
13	M	69	7	858	Very high protein (12 mg/ml)	Unclear, CO	AD	**X**
14	F	75	25	105,111	Extreme high tau	Unclear, MCI	MCI, TBI, vomiting, diarrhea	**X**
15	M	75	11	2,298		CO	AD	**N**
16	F	85	15	1,576	After breakfast	Unclear, CO	AD, vascular	**X**
17	F	80	88	377		MCI	MCI	**Y**
18	F	71	18	301		MCI/ADD	MCI	**Y**
19	F	81	52	27,682	Very high tau	Unclear, MCI	MCI	**X**
20	F	74	14	387		CO	CO	**Y**
21	F	79	16	825		CO	MCI	**N**

AD, Alzheimer’s disease dementia; CO, control or depression; F, female; M, male; MCI, mild cognitive impairment (including beginning AD dementia); N, diagnosis not correct; X, not clear and not included; Y, diagnosis correct.

## Discussion

In this present study, we could show that total tau and pTau181 can be reliably detected in human saliva, while Aβ-42 and –40 were below the detection limit. A significant increase of pTau181 is shown for patients with MCI and a decrease of total tau for patients with AD dementia, which could make them useful as biomarkers, especially in combination with the very sensitive Lumipulse technology.

### The heterogeneity of saliva

Saliva is a human fluid that is easily applicable, without any ethical concerns, but is still underrecognized as a source of AD biomarkers. The saliva proteome contains approx a total of 2,300 proteins and 27% are identical to plasma proteins (Loo et al., 2010). Saliva is the fluid that bathes the mouth and oral cavity and is made and secreted from the salivary glands (parotid, submandibular, sublingual, and minor salivary glands) which are directly innervated *via* cranial nerves (facial nerve, glossopharyngeal nerve). Furthermore, it consists of non-salivary components gingival crevicular fluid, nasal and bronchial secretions, serum and blood derivatives from wounds, desquamated epithelial linings, food components, and micro-organisms in the oral cavity (Kaufman and Lambster, 2002; Loo et al., 2010). Whole saliva is composed of water, peptides, and proteins, including hormones and enzymes, sugars, lipids, and electrolytes. The saliva metabolome appears to be comparable to the human serum and CSF metabolome in terms of chemical complexity and the number of compounds (Dame et al., 2015). This is consistent with the data from previous studies that showed that compounds found in human saliva are usually found in human blood, albeit at different concentrations (Takeda et al., 2009). Our data clearly show that saliva displays a very high variability even if it was collected the same way and also within the same group. First, the total protein content varied between 9,000 and 160 μg/ml. Second, when the saliva was applied onto an HPLC with EC detection (+ 0.55V), then the chromatogram was also very heterogeneous, sometimes with a few clear peaks, sometimes with peaks detectable, and sometimes there appeared a broad smear within the first 6 min, which did not allow to detect NE (rt = 5 min). This clearly shows that saliva can be a heterogenous fluid and this must be considered when saliva should be used as a fluid for biomarker detection.

### Saliva and beta-amyloid

As mentioned, there were some studies showing that saliva Aβ−42 could be a potential biomarker for AD and is enhanced in saliva (Bermejo-Pareja et al., 2010; Lee et al., 2017; Sabbagh et al., 2018; Gleerup et al., 2019), while others could not even detect it (Shi et al., 2011). There is also a report showing that amyloid-precursor protein (APP), the precursor protein of Aβ-42, was found to be expressed in human salivary epithelial cells (Oh and Turner, 2006). Our results showed that Aβ was close or below to the detection limit in most of the saliva samples. During the revision, we performed additional experiments and found that beta-amyloid cannot be recovered from the Salivettes and binds probably to the cotton. This was unfortunate, as the whole study was performed with Salivettes. We showed in a subsequent additional experiment with healthy volunteers, that, indeed, Aβ-40 and –42 can be detected in native saliva (spitting directly into tubes), and thus we recommend not to use Salivettes for such biomarker studies.

### Saliva and tau

In the present study, we show that saliva total tau and pTau181 are detectable and altered in patients with AD dementia and MCI. We found lowest levels of total tau in patients with AD dementia and significantly or slightly increased levels of pTau181 in patients with MCI and AD dementia. Our results are in agreement with those of Lau et al. (2015) who found a higher expression of phospho-tau in patients with AD compared to controls. In contrast to Pekeles et al. (2018), we found no significant changes in the tau/pTau181 ratio in our study sample. Our study, however, could show for the first time that tau-markers are altered already in MCI, which is associated with increased risk for conversion to AD dementia.

Therefore, our results might make an important contribution to the current understanding of biomarkers in the early clinical stages of AD. In support of this, our blinded study was able to differentiate between MCI and AD dementia with an accuracy of over 70%. However, other authors argued that a large variation in the AD salivary tau levels limits the utility of tau as a clinical biomarker. We agree with this assumption, but note that prior studies showed high heterogeneity in saliva collection and analyzing methods. Our present study shows that the very sensitive Lumipulse technology is very potent to determine saliva total tau and pTau181 in fresh and frozen samples.

While total tau and phosphorylated tau in CSF originate from the brain, the origin of tau in saliva is not yet sufficiently clear. It has been suggested, that tau could be secreted from the innervating cranial nerves into the salivary glands (Kim et al., 2010), is derived from ultrafiltration from blood, or comes directly from epithelial cells. Supporting the latter option, tau mRNA was reported to be highly expressed in salivary glands (Conrad et al., 2002). This can lead to the assumption that tau comes directly from the salivary glands, which is also supported by our preliminary data with mouse salivary glands. Our data show that tau is, indeed, present in human saliva, and it was surprising to see that Lumipulse detects relatively high levels of tau (300–700 pg/ml) which are comparable to the tau levels in CSF. In addition, we detected some saliva samples, where tau was in the far ng/ml range, which means 1,000 × higher tau levels. This underlines that Lumipulse assay is very sensitive and accurate and we trust that the assay detects real tau; however, we cannot completely exclude some forms of small measuring inaccuracies. It needs to be noted that the Lumipulse technology has been validated for CSF and not saliva. Moreover, all the extreme tau levels could be related to patients with recent inflammation of salivary glands or bleedings into the mouth. More work is definitely necessary to investigate this issue.

In a preliminary experiment, we aimed to analyze saliva by Western blot (using a pool of 50 ml saliva from a healthy volunteer), but the detection levels were extremely low, and the data suggested tau fragments of approx. 30 kDa (data not shown). This needs a better investigation with more sensitive methods, but could fully agree with the very important work from Meredith et al. (2013). They show that multiple N-terminal and mid-domain fragments of tau were detected in pooled CSF with apparent sizes ranging from 20 kDa to approx. 40 kD. The pattern of tau fragments in AD and control samples was similar. They (Meredith et al., 2013) also did not detect full-length tau and C-terminal fragments in their CSF samples. Definitely, more work is necessary to study the metabolism and stability of the tau protein and the functional role of smaller fragmented tau forms.

### Limits of the study

The blinded verification study already highlights several limits of the study and of saliva as a source of human fluid biomarkers. (1) First, we found that the saliva protein showed a strong heterogeneity from very high to very low protein values. Neither the laboratory nor the clinicians are able to verify that the saliva collection was done very accurately. Possible bias could be the consumption of food before saliva collection or that patients had the Salivette for less than 2 min in the mouth. (2) Second, we found some samples with very high saliva tau, in the far ng/ml range. This is surprising and should be verified by other methods. We could correlate some high saliva tau levels with acute brain injury or inflammation of the glands. Alternatively, we cannot exclude bleeding of the teeth or periodontitis, and thus a dentist could give advice in future studies. (3) Unfortunately, the use of Salivettes was not optimal as beta-amyloid could not be recovered. Indeed, there is no international consensus on saliva collection procedure, stability, origin, inflammatory processes in the mouth, and salivary flow of salivary glands. This all needs to be tested in future studies. (4) And finally, in saliva, a functional role of tau is still largely unexplored. It will be necessary to conduct more research in humans, or in mouse models, including tau transgenic mice.

In conclusion, our data show that total tau and pTau181 are reproducible and detectable in saliva and total tau is decreased in AD dementia, while pTau181 is increased in MCI. We suggest that the very sensitive Lumipulse technology could become an interesting tool to establish saliva tau detection in AD. However, saliva is a heterogeneous fluid and the collection process needs to be established and verified in order to provide stable values. We recommend not using Salivettes for saliva sample collection.

## Data availability statement

The raw data supporting the conclusions of this article will be made available by the authors, without undue reservation.

## Ethics statement

The study was approved by the Ethics Committee of Medical University of Innsbruck. Written informed consent to participate in this study was provided by the participants’ legal guardian/next of kin.

## Author contributions

CH designed the study, wrote the manuscript, and acquired funding. JM collected saliva samples and diagnosed patients. MD collected saliva samples and diagnosed patients for the blinded study. All authors contributed to the article and approved the submitted version.

## Footnotes

1
https://www.fujirebio.com/en/products-solutions/lumipulse-g600ii
